# DLC1 suppresses NF-κB activity in prostate cancer cells due to its stabilizing effect on adherens junctions

**DOI:** 10.1186/2193-1801-3-27

**Published:** 2014-01-14

**Authors:** Veenu Tripathi, Nicholas C Popescu, Drazen B Zimonjic

**Affiliations:** Laboratory of Experimental Carcinogenesis, National Cancer Institute, National Institutes of Health, 37 Convent Drive, MSC 4262, Bethesda, Maryland 20892 USA

**Keywords:** DLC1, NF-kB, Rho, Adherens junction (AJ), Prostate cancer

## Abstract

DLC1 (Deleted in Liver Cancer 1) gene encodes a RhoGTPase-activating protein (RhoGAP), which exerts most of its tumor suppressor functions through suppression of small Rho GTPases proteins RhoA, RhoB, RhoC and to some degree Cdc42, but not Rac. RhoGTPases are implicated in NF-κB activation in highly invasive prostate carcinoma (PCA), with consequences on cell proliferation, survival and metastatic capacity. Here we demonstrate that DLC1 transduction in two androgen-independent (AI) and highly metastatic PCA cell lines negatively regulates NF-κB activity in a GAP- and α-catenin-dependent manner. Expressed DLC1 protein suppresses the phosphorylation of NF-κB inhibitor, IκBα, causes its relocation from membrane ruffles into cytoplasm and attenuates its ubiquitination and subsequent degradation. DLC1-mediated NF-kB suppression and its effects are comparable to NF-κB inhibition using either shRNA knockdown or peptide inhibitor. Expression of transduced DLC1 suppressed the expression of NF-κB mediated genes. Such effects were found to be reliant on presence of calcium, indicating that the observed modifications are dependent on, and enabled by DLC-mediated stabilization of adherens junctions. These results expand the multitude of DLC1 interactions with other genes that modulate its oncosuppressive function, and may have potential therapeutic implications.

## Background

Metastatic prostate cancer (PC), resistant to conventional therapies, hormone deprivation and ablation, is one of the leading causes of death of men in United States. In about one third of post-prostatectomy patients, tumor cells reappear as local or distant metastasis, develop hormone refractoriness and acquire increasingly more aggressive phenotype. A growing body of evidences shows that a transcriptional factor NF-κB is constitutively activated in primary PC (Suh et al. 2002), and plays an important role in tumor invasion, metastasis, angiogenesis, and acquisition of chemotherapy resistance (Sweeney et al. 2004; Catz and Johnson 2001).

Although commonly called NF-kB, it is in fact a group of related homo- and heterodimeric transcription factors composed of five members of the Rel/NF-κB family of proteins - RelA (p65), RelB, c-Rel, NF-kB1 {p50) and NF-kB2 (p52) – all of which contain a conserved Rel homology region and C– and N-terminal domains responsible for dimerization and DNA binding, respectively (Hoffmann et al. 2003). Activation of NF-κB occurs when its specific inhibitor from the Ik-B family of proteins gets phosphorylated (and subsequently degraded) by either tripartite (IKKα, IKKβ, IKKγ) or homodimeric (IKKα, IKKα) I-kB kinase (IKK) complex (Ghosh and Karin 2002; Luo et al. 2005). Upon activation NF-κB relocates from cytoplasm to nucleus where, after some additional molecular modifications, it binds to promoter region of numerous genes involved in inflammatory response, cell growth, apoptosis and differentiation. Increased action of IKK complex appears to be in the root of constitutive NFκB activity in PC (Palayoor et al. 1999; Pajonk et al. 1999), which affects expression of genes like cyclin D1, c-myc and Bcl-2 that drive cellular proliferation or confer resistance to apoptotic signals. NF-κB-mediated expression of genes like IL-8, VEGF, MMP9, vimentin, uPA and uPA receptor, further contribute to the development of aggressive PCA (Karin et al. 2002).

On the other side, among multiple factors implicated in the initiation and progression of PC (Xu et al. 2002; Albany et al. 2011; Hsieh et al. 2001) tumor suppressor gene DLC1, a member of RhoGAP family of genes, stands out, as it has been shown to be down-regulated or absent not only in PC, but in various other solid tumors and hematological malignancies (Guan et al. 2006; Durkin et al. 2007; Ullmannova et al. 2009). Introduction of DLC1 in highly metastatic PCA cells suppressed proliferation, invasiveness and anchorage-independent growth and restored response to apoptotic signaling (Guan et al. 2008). Conversely, DLC1 silencing in prostate epithelial cells promoted pro-angiogenic responses through up-regulation of vascular endothelial growth factor (VEGF), accompanied by the accumulation of hypoxia-inducible factor 1α and its nuclear localization (Shih et al. 2010).

The rationale for undertaking this study was based on evidence showing that NF-κB is constitutively activated in PC cells, is a major transcription factor involved in metastasis to bone, and, importantly, DLC1expression suppresses proliferation of PC cells *in vitro*, and their growth in nude mice (Andela et al. 2003 Guan et al. 2008). We used metastatic, androgen-independent PCA cell lines C4-2-B2 and PC-3, deficient in DLC1 expression but with high NF-κB activity, to examine the relationship between NF-κB and DLC1 and showed that in these cells restoration of DLC1 expression inhibited the phosphorylation (i.e. activation) of NF-κB in the α-catenin- and GAP-dependent manner.

## Results

### DLC1 suppresses NF-κB activity in prostate cancer cells

**Figure 1:**
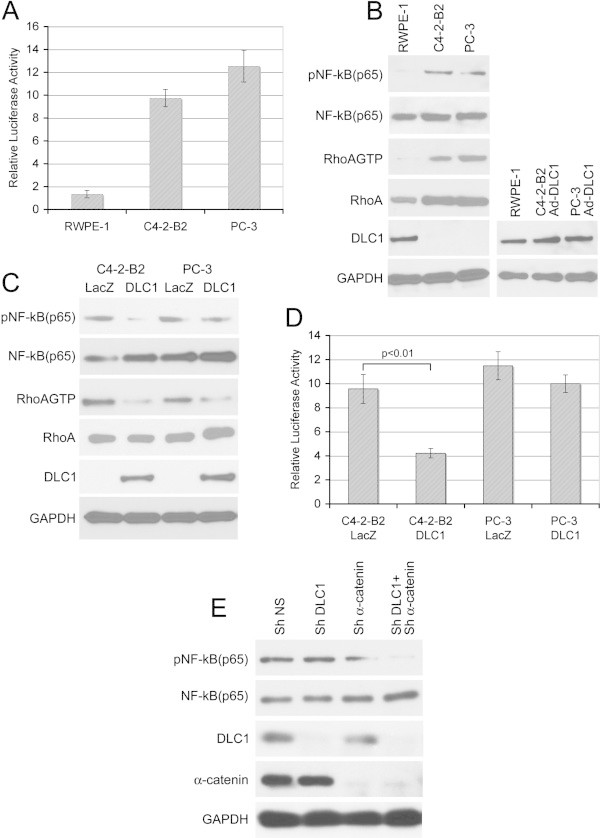
**DLC1 suppresses NF-κB activation. (A)** Relative luciferase activity of NF-κB reporter in RWPE-1, C4-2-B2 and PC-3 cells, **(B)** Western blot showing the level of DLC1, pNF-κB (p65), NF-κB (p65), RhoAGTP, and total RhoA in RWPE-1, C4-2-B2 and PC-3 cells and level of DLC1 in RWPE-1 and Ad-DLC1 transduced (50MOI) C4-2-B2 and PC-3cells. **(C)** Western blot showing level of phosphorylated p65 subunit of NF-κB in the Ad-Lac Z and Ad-DLC1 transduced C4-2-B2 and PC-3 cells. **(D)** Relative luciferase activity of NF-κB reporter in AD-LacZ and DLC1 transduced C4-2-B2 and PC-3 cells. All values are means ± SD of three independent counts. **(E)** Western blot showing the level of phosphorylated p65 subunit of NF-κB in DLC1- and α-catenin-knock down RWPE-1 cells.

When compared to normal, immortalized, DLC1-positive prostate epithelial cell RWPE-1, metastatic PCA cells C4-2-B2 and PC-3 showed (Figure 1A, B) lack of DLC1 expression, higher levels of active Rho, and significantly higher levels of NF-κB activation, as measured by phosphorylation of p65 subunit of NF-κB. Transduction of DLC1 into C4-2-B2 cells, and restoration of its expression to the level comparable to that in RWPE-1 (Figure 1B), reduced activation of NF-κB as demonstrated by both p65 phosphorylation (Figure 1C) and by luciferase reporter assay (Figure 1D). On the other hand, re-expression of DLC1 in PC3 cells did not significantly affect NF-κB activation (Figure 1C, D), although a DLC1 was suppressing active RhoA in both cells (Figure 1C). Knocking down of both DLC1 and α-catenin in RWPE-1 cells suppresses the phosphorylation of p65 subunit of NF-κB and, thus, underscores the role of DLC1 and α-catenin in NF-κB activity (Figure 1E).

### DLC1-mediated suppression of NF-κB is α- catenin dependent

**Figure 2:**
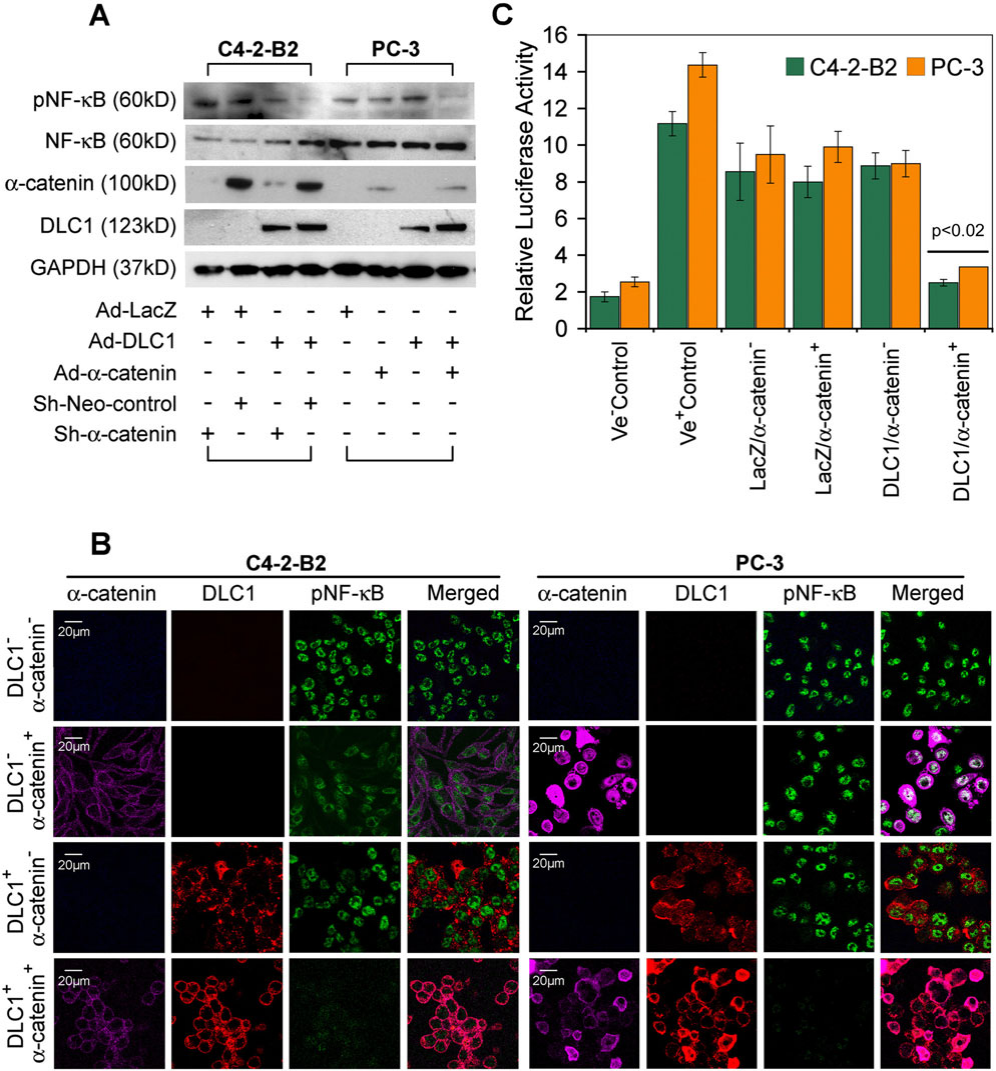
**Both DLC1 and α –catenin expression are required to regulate NF-κB activity. (A)** Western blot of total and phosphorylated NF-κB from cell lysates of C4-2-B2 (transduced and/or stably transfected with Ad-LacZ, Ad-DLC1, Ad-DLC1 + sh-Neo or Ad-DLC1 + sh-α-catenin) and PC- 3 cells (transduced with Ad-LacZ, Ad-DLC1 orAd-DLC1 + Ad-α -catenin). **(B)** Immunostaining of the above described transduced and stable transfected C4-2-B2 and PC- 3 cells with anti-phospho NF-κB, anti-DLC1 and anti-α -catenin antibodies. Scale bar = 20 μm. **(C)** C4-2-B2, and PC-3 cells were transfected with NF-κB reporter vector and luciferase activity was measured with dual luciferase assay. All values are means ± SD of three independent counts.

Since PC3 cells, in addition to absence of DLC1 expression, also lack expression of α- catenin, which is abundantly expressed in C4-2-B2 cells, we hypothesized that α-catenin might be a “missing link” in DLC1-mediated inhibition of NF-κB activation of PC3 cells. We, therefore, probed extracts of C4-2-B2 and PC-3 cells with anti-phospho-p65 antibody, to see if α-catenin status affects p65 phosphorylation and activation of NF-κB. In both cell lines simultaneous expression of DLC1 and α-catenin was associated with lower levels of p65 phosphorylation, indicative of reduced NF-κB activation (Figure 2A). Confocal imunnofluorescence analysis of C4-2-B2 and PC-3 cells (Figure 2B) also showed that presence of either DLC1 or α-catenin did not affect the nuclear phosphorylated NFκB, but that co-expression of DLC1 and α-catenin reduced the amount of active NFκB below the level of detection. NF-κB luciferase reporter gene assay (Figure 2C) provided additional confirmation that both DLC1 and α-catenin are required for the maximum suppression of NFκB activation; used alone neither the full-length DLC1 nor α-catenin were not able to produce a statistically significant effect.

### RhoGAP activity and interaction with α-catenin is required for NF-κB down regulation

**Figure 3:**
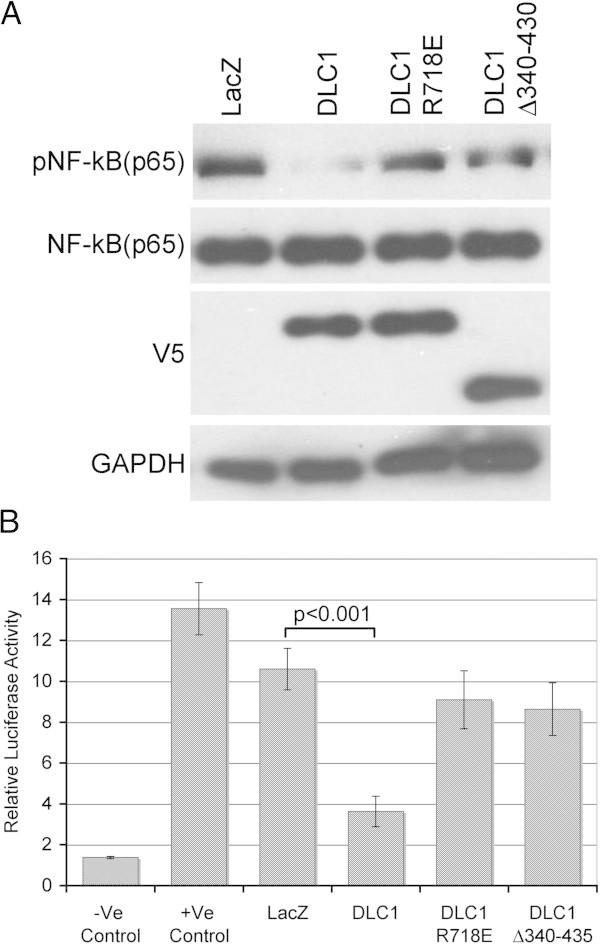
**Suppression of NF-κB activity is GAP- and α –catenin-dependent (A) Western blot of pNF-κB (p65), NF-κB (p65) and DLC1 (V-5) expression in C4-2-B2 cells transduced with Ad-Lac Z or Ad-DLC1 and stably transfected with either DLC1 GAP-mutated (R718E) or α -catenin binding deficient constructs (Δ340-435). (B)** Dual Luciferase reporter assay for NF-κB activity in above-mentioned C4-2-B2 cells. All values are means ± SD of three independent counts.

To see if DLC1-mediated inhibition of NF-κB activity was RhoGAP-dependent, C4-2-B2 cells were either transduced with wild-type DLC1, or transfected with DLC1 GAP mutant R718E. In the later case, stable clones were selected using G148. Subsequent comparison of NF-κB p65 subunit phosphorylation in aforementioned GAP-competent and GAP-incompetent C4-2-B2 cells showed no reduction in p65 phosphorylation and, therefore, no inhibition of NF-κB activation in cells bearing mutant GAP (Figure 3A). In addition, cells transfected with DLC1 deletion mutant Δ340-435, lacking the critical α-catenin-binding region, also were not able to suppress the phosphorylation and activation of NF-κB (Figure 3A). Luciferase reporter gene activation assay confirmed these observations (Figure 3B), which, together, revealed that DLC1-mediated NF-κB regulation depends on both the DLC1 RhoGAP activity, and on its ability to effectively bind α-catenin.

### DLC1-mediated adherens junctions stabilization is responsible for inhibition of NF-κB activity

**Figure 4:**
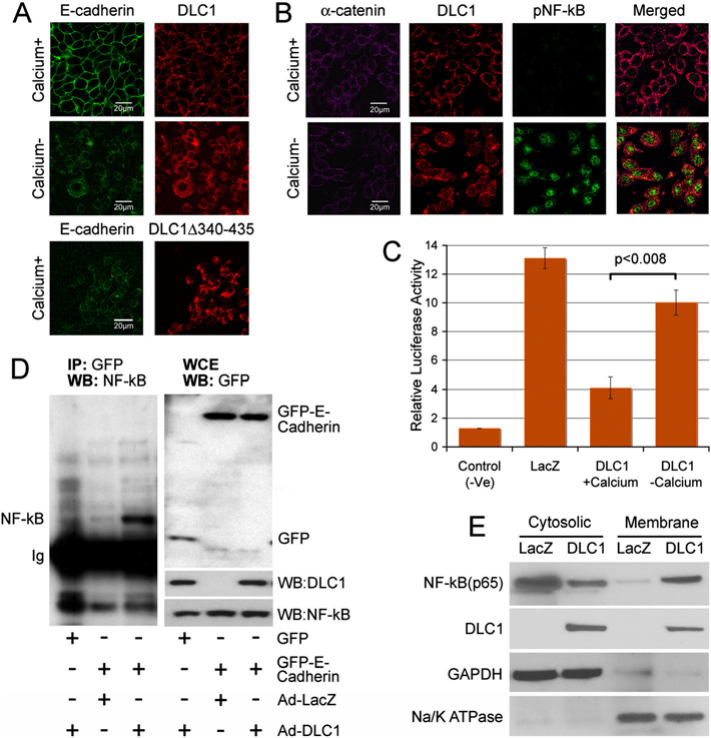
**DLC1 affects NF-κB activity is dependent on AJs stability. (A)** C4-2-B2 cells, transduced with Ad-DLC1 or transfected with deletion mutant DLC1 Δ340-435 (lacking the α-catenin-binding region), were grown in media with or without calcium, and immunostained for E-cadherin and DLC1. Scale bar = 20 μm. **(B)** C4-2-B2 cells transduced and stably transfected with Ad-DLC1 and sh-α -catenin were grown in media with and without calcium for 24 hours, and stained for phospho NF-κB, DLC1 and α -catenin. Scale bar = 20 μm. **(C)** Luciferase reporter assay confirms increased NF-κB activity in C4-2-B2 cells grown in calcium-negative media. **(D)** Cell extracts from C4-2-B2 cells transduced and stably transfected with Ad-DLC1 + GFP (lane 1), Ad-LacZ + GFP-E-cadherin (lane 2) or Ad-DLC1 + GFP-E-Cadherin, were immunoprecipitated with anti-GFP antibodies and western blotted with anti-NFκB-antibodies. Result of the whole cell extract is shown in the right. **(E)** Membrane and cytosolic fractions of NF-κB in the LacZ and DLC1 transduced C4-2-B2 cells.

The integrity of adherens junctions (AJ) is affected by availability of calcium ions (Ca^2+^); depletion of Ca^2+^ disrupts AJs as shown by E-cadherin staining of DLC1-transduced C4-2-B2 cells, and cells with DLC1 deletion mutant Δ340-435, lacking the critical α-catenin-binding region (Figure 4A). Interestingly, such cells with compromised AJ integrity show activation of NF-κB, as demonstrated by anti-pNF-κB staining in absence of Ca^2+)^ (Figure 4B) and confirmed by luciferase reporter assay (Figure 4C). To examine if DLC1-mediated stabilization of AJs would reduce NF-κB activity by enabling its binding to AJ’s proteins, primarily E-cadherin, C4-2-B2 cells transduced with either LacZ or DLC1, were additionally transfected with GFP E-cadherin (Figure 4D right), their respective cell extracts immunoprecipitated with anti-GFP, and those immunoprecipitates probed with anti-NF-κB antibodies. When compared to DLC1-negative LacZ-transduced cells, DLC1-positive cells exhibited increased amounts of NF-κB associated with E-cadherin (Figure 4D left) and reduced NF-κB activity. Furthermore, analysis of membrane and cytosolic fractions of LacZ- or DLC1-transduced C4-2-B2 cells, showed a pronounced shift in NF-κB distribution from cytosole to membrane in DLC-positive cells (Figure 4E), indicating that DLC1-mediated stabilization of AJs plays an important role in modulating NF-κB activity.

### DLC1 inhibits IκBα localization to membrane ruffles and proteasomal degradation

**Figure 5:**
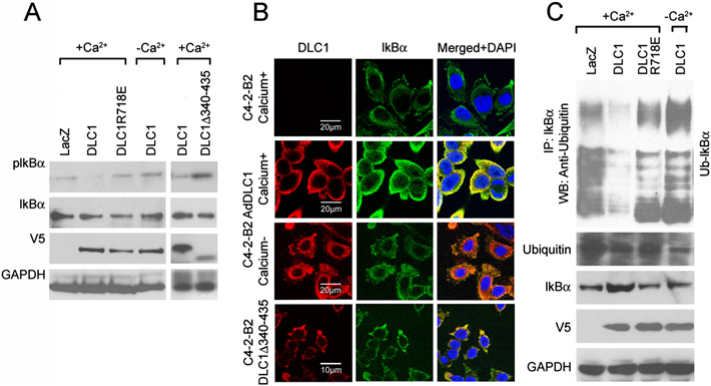
**DLC1 expression causes relocation and suppresses phosphorylation and ubiquitination of IκBα. (A)** Western blots of phospho-IκBα and total IκBα in Ad-LacZ-, Ad-DLC1-transduced or DLC1 deletion mutant ΔD340-435-transfected C4-2-B2 cells. **(B)** IκBα localization in Ad-LacZ-, Ad-DLC1-transduced or DLC1 deletion mutant ΔD340-435-transfected C4-2-B2 cells in the presence and absence of calcium. Scale bars 20 μm and 10 μm, respectively. **(C)** Ubiquitination assay of IκBα of proteasome inhibitor MG132 treated Ad-LacZ and Ad-DLC1 transduced C4-2-B2 and DLC1R718E transduced cells in the presence and absence of calcium.

To further explore the mechanism through which DLC1 modulates NF-κB activity, we examined phosphorylation of NF-κB’s specific inhibitor, IκBα, a molecular modification instrumental for the release of active NF-κB to nucleus. As shown in Figure 5A, phosphorylation of IκBα was decreased in the presence of DLC1. Use of DLC1 deletion mutant Δ340-435 caused no effect on pIkB phosphorylation, thus affirming the role of this region of DLC1 in the signaling.

Confocal microscopy analysis of IκBα cellular distribution showed that in DLC1-negative C4-2-B2 cells the inhibitor was localized to the membrane ruffles. Transduction and expression of DLC1 was followed by IκBα distribution throughout the cytoplasm but, interestingly, Ca^2+^ depletion reversed that process causing IκBα to withdraw to membrane again (Figure 5B). Expression of DLC1 deletion mutant ΔD340-435 lacking the α-catenin-binding region, was not able to completely relocalize IκBα to the cytoplasm, and certain amount of IκBα remained present in the membrane ruffles (Figure 5B).

Ubiquitination assay of IκBα showed that the expression of wild-type DLC1 attenuated ubiquitination of IκBα, whereas GAP-incompetent DLC1 mutant R718E failed to produce such an effect (Figure 5C). Here again elimination of calcium from culturing media reversed the effects of DLC1 expression, i.e., increased the ubiquitination of IκBα, suggesting that DLC1-mediated stabilization of AJs, and spatial distribution of both IκBα and NF-κB are two major factors affecting NF-κB signaling.

### Colony formation, invasiveness and apoptosis of metastatic prostate carcinoma cells are affected similarly by DLC1-mediated NF-κB suppression, sh(p65)NF-κB silencing or pharmacological down-regulation of NF-κB

**Figure 6:**
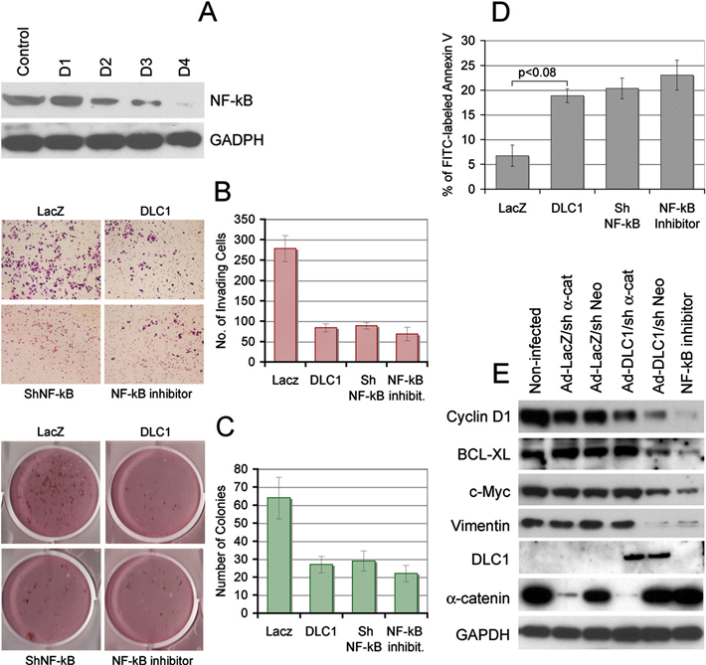
**DLC1-mediated NF-κB suppression shows effects similar to sh(p65)NF-κB silencing or pharmacological down regulation of NF-κB. (A)** Western blot showing NF-κB (p65) knock down, D1, D2, D3 and D4 are different clones. Clone D4 was taken for study. Microphotographs and/or quantitative valuation of Invasion Assay **(B)** Colony Formation **(C)** and Apoptosis Assay **(D)** of C4-2-B2 cells transduced with Lac Z or DLC1, stably transfected with shNF-κB or treated with NF-κB inhibitor (Peptide inhibitor 150 μM). **(E)** Western blot analysis of NF-κB-induced genes in cell extract from C4-2-B2 transduced and stably transfected with Ad-LacZ + sh-α-catenin, Ad-LacZ + sh-Neo, Ad-DLC1 + sh-α-catenin, Ad-DLC1 + sh-Neo, or NF-κB peptide inhibitor (150 μM).

To better understand the magnitude and the consequences of DLC1-mediated suppression of NF-κB activation, DLC1-negative C4-2-B2 cells were either transduced by DLC1 gene, or treated by shRNA (targeting p65 subunit of NF-κB), or by pharmacological NF-κB peptide inhibitor. Use of sh(p65)NF-κB significantly reduced NF-κB expression on protein level (Figure 6A) whereas use of 150 μM peptide inhibitor completely abolished NF-κB transcriptional activity. Expression of DLC1 protein reduced colony formation efficiency, and invasion capability of C4-2-B2 cells, to the levels similar to one caused by sh(p65)NF-κB or by chemical NF-κB peptide inhibitor (Figure 6B, C). The same pattern was observed in apoptosis assay where DLC1 effect was comparable to the ones caused by NF-κB inhibitors (Figure 6D). In addition, expression of several NF-κB-inducible genes with roles in cell proliferation, invasion and apoptosis - such as cyclin D1, c-Myc, Bcl-xL and vimentin - was notably suppressed by simultaneous co-expression of DLC1 and α-catenin, in a pattern similar to the effect of NF-κB peptide inhibitor (Figure 6E), which, altogether, further underscores the significance of DLC1 in NF-κB- mediated signaling.

## Discussion

This study demonstrates that DLC1 expression in androgen-independent and highly metastatic PCA cells C4-2-B2 and PC-3 with constitutive activation of NF-κB (Gasparian et al. 2002) negatively regulates NF-κB activity in a GAP- and α-catenin-dependent manner. DLC1 suppresses the phosphorylation of NF-κB inhibitor, IκBα, causes its relocation from membrane ruffles, and attenuates its ubiquitination and subsequent degradation. DLC1-mediated NF-kB suppression and its consequences were comparable to NF-κB inhibition using either shRNA knock down or NF-κB peptide inhibitor. In addition, DLC1 transduction suppressed the expression of NF-κB mediated genes. These effects were found to be reliant on presence of calcium, which indicates that the observed modifications are dependent on, and enabled by DLC1-mediated stabilization of adherens junctions (Tripathi et al. 2012).

Constitutive activation of NF-κB pathway in prostate, lung, liver and breast cancers (Gasparian et al. 2002; Chiao et al. 2002; Biswas et al. 2004), correlates with the expression of several genes involved in immune and inflammatory responses, neoangiogenesis, increased cell proliferation, epithelial-to-mesenchymal transition (EMT) and in acquired resistance to apoptosis (Huber et al. 2004; Pikarsky et al. 2004; Julien et al. 2007; Pickering et al. 2007; Hafeez et al. 2008). NF-κB has been shown to promote breast cancer metastasis (Park et al. 2007), and appears to be a major regulator of prostate cancer cells metastasis to bone (Andela et al. 2003). Inhibition of NF-κB activity in tumor cell lines increases their sensitivity to chemotherapeutic drugs and radiation (Amit et al. 2003; Tapia et al. 2007). Transcriptional activation of NF-κB is effectively induced – independent of RasGTPase and Raf-1 kinase - by members of Rho family of small GTPases, that include RhoA, Cdc42 and Rac1 (Hodge et al. 2003; Perona et al. 1997; Cammarano and Minden. 2001; Gnad et al. 2001). Conversely, blockage of Rho pathways leads to suppression of NF-κB activity (Segain et al. 2003). This relationship is becoming increasingly important in deciphering regulatory underpinning of one of the crucial elements of metabolic reprogramming of cancer cell – it’s greatly elevated glutamine metabolism - where, apparently, activation of the predominant isoform of mitochondrial enzyme glutaminase is substantially influenced by concomitant RhoGTPase signaling and NF-kB activation (Wilson et al. 2013).

Main function of DLC1 gene-encoded protein is inactivation of Rho family of small GTPases (Durkin et al. 2007; Guan et al. 2008) by catalyzing the conversion of active GTP-bound, into GDP-bound inactive form, and thus blocking Rho pathway. The product of DLC1 gene was shown to inactivate RhoA, RhoB, RhoC and to some degree Cdc42, but not Rac (Zimonjic and Popescu 2012). DLC1 is frequently down regulated in prostate cancer by either epigenetic modifications or deletion (Guan et al. 2006) and its genomic under-representation at chromosome 8p is associated with aggressive form of prostate cancer (Matsuyama et al. 2001). Both C4-2-B2 and PC-3 cells are cancer cells and, as compared to normal cells such as RWPE-1, exhibit higher level of RhoA. High level of total RhoA could affect Rho activity only if RhoGAPs such as DLC1 - for which these cells are negative - are absent. Otherwise, high total RhoA would not have much effect on overall Rho activity*.* Therefore, DLC1-mediated suppression of NF-κB activation could be a reflection of disruption of Rho signaling pathway by one powerful RhoGAP, even more so given that the results presented above demonstrated the dependence of suppression on DLC1’s GAP activity. Yet, the fact that re-expression of DLC1 alone was not sufficient to affect NF-κB activation in α-catenin-negative PC3 cells, as opposed to α-catenin positive C4-2-B2 cells, points to a more complex mechanism.

Loss of α-catenin in cancer cells results in increased cell proliferation and resistance to apoptosis (Liu et al. 2007; Lien et al. 2006) whereas variations in availability of either α-catenin or β-catenin were shown to influence functional status of NF-κB (Deng et al., 2002; Kobielak and Fuchs 2006; Solanas et al. 2008). Increased activation of NF-κB in C4-2-B2 and PC3 cells occurred in absence of either DLC1 or α-catenin - or both of them - whereas only the simultaneous expression of DLC1 and α-catenin was effective in suppressing the NF-κB activity, thus portraying DLC1’s GAP function as necessary, but not enough. However, just removal of calcium from chemical environment was sufficient to cancel the joint DLC1-α-catenin suppression of NF-κB activation.

Calcium is instrumental for formation and integrity of contact points between epithelial cells – adherens junctions (AJ) - whose major molecular component is E-cadherin, which maintains the connection to the actin cytoskeleton through interaction with catenins (Wheelock and Johnson 2003), and whose loss leads to up-regulation of NF-κB activity (Kuphal et al. 2004). Conversely, stable association of NF-κB with AJ’s proteins, primarily E-cadherin, reduces its activity (Solanas et al. 2008; Kuphal et al. 2004). Apparently, such immobilization of NF-κB is enabled by β catenin, which acts as possible link between p65 subunit of NF-κB and adherens junctions (Solanas et al. 2008; Kuphal et al. 2004). DLC1 contributes to AJ’s stabilization through its interaction with E-cadherin via α-catenin or by inducing E-cadherin expression (Tripathi et al. 2012; Tripathi et al. 2013). One of the consequences of increased AJ’s stability is down-regulation of RhoGTPases (Asnaghi et al. 2010). As our aforementioned analysis of membrane and cytosolic cellular fractions showed, DLC1 expression resulted in higher rate of association of p65 subunit with the membrane, thus signaling increased membrane localization of NF-κB, which coincided with its reduced activity.

Inhibition of NF-κB activity in human prostate cancer cells suppresses invasion, metastasis, and neoangiogenesis (Huang et al. 2001). Our results show that a major NF-κB inhibitor, IκBα, whose IKK-mediated phosphorylation, ubiquitination and subsequent degradation takes place in membrane ruffles (Boyer et al. 2004) is indeed, localized in membrane ruffles of DLC-1 negative cells – but is relocated into cytoplasm and, thus, rescued from proteasomal degradation in cells with restored DLC1 expression. Although IκBα physically interacts with cytoskeleton-associated protein (Crepieux et al. 1997), we do not have any evidence that DLC1 and IκBα proteins directly interacts with each other. The fact that such a process is contingent on presence of calcium, reaffirms that the stability of AJs, resulting from intricate molecular interactions between DLC1, α-catenin and E-cadherin, appears to play a major role in regulating NF-κB activity.

## Conclusions

This study provides new evidences that tumor suppressor gene DLC1, through its RhoGAP activity, affects the activation of NF-κB and, thus, modulates the complex signal transduction pathways, which associate with inflammatory response and cancer progression. It expands the known DLC1 role and opens the prospect that DLC1 introduction, or the inhibition of downstream pathways activated by DLC1 deficiency, could sensitize chemotherapy-resistant metastatic cancer to various pharmacological drugs.

## Methods

### Cell lines and culture conditions

C4-2-B2 cell line was purchased from ViroMed, lab Inc (Minneapolis, MN) and cultured in T-medium (Invitrogen, San Diego, CA) containing 10% FBS. PC-3 and RWPE-1 cells lines were purchased from American Type Culture Collection (Rockville, MD). PC-3 was cultured in RPMI 1640 medium (Invitrogen, San Diego, CA) and RWPE-1 was cultured in keratinocyte medium (Invitrogen, San Diego, CA) supplemented with Epithelial Growth Factor (Invitrogen, San Diego, CA) and Bovine Pituitary Extract (Invitrogen, San Diego, CA). All cell cultures were grown in a humidified CO_2_ incubator at 37°C.

### Plasmids and transfections

For stable knock down of NF-κB (P65 subunit), four respective SureSilencing shRNA plasmid vectors (KH01812P, SA Biosciences, Frederick, MD), containing puromycin-resistance gene were transfected using lipofectamine 2000 (Invitrogen, San Diego, CA). The media containing puromycin was changed after 48 hours. For stable transfection of α-catenin (KH00646N with neomycin) short hairpin RNA (shRNA) and for DLC1 knock down (KH00438P with Puromycin), SureSilencing shRNA plasmid vectors (SA Biosciences, Frederick, MD) were transfected using Lipofectamine 2000, and medium containing appropriate antibiotics was changed after 48 h. Two weeks after transfection, single colonies were picked up and expanded. Stable clone for DLC1 R718E was made using G418 resistance. An adenovirus encoding either DLC1 cDNA or LacZ was prepared and transduced as previously described (Guan et al., 2008). Adenovirus encoding α-catenin was purchased from Vector Biolabs (Philadelphia, PA).

### Immunoprecipitation and western blotting

Cells were transduced or transfected with various DLC1 constructs, α-catenin, and/or GFP-cadherin and lysed with NP-40 lysis buffer (BioSource, Camarillo, CA) containing Protease Inhibitor Cocktail (Sigma, St Lois, MO). Centrifugation was done for 20 min at 12,000 × g to produce lysate for western blot or immunoprecipitation. Anti-GFP antibody was added into precleared cell lysates and incubated overnight at 4°C. Protein G-Sepharose slurry (Zymed Inc., San Francisco, CA, USA) was added and incubated for additional 2–3 hours. Immunoprecipitates were washed and loaded on 4–12% SDS-PAGE gels (Invitrogen, Carlsbad, CA), transferred to nitrocellulose membranes (Invitrogen, Carlsbad, CA) and western blotted with anti NF-κB antibody. Proteins were visualized by horseradish peroxidase–conjugated secondary antibodies and chemiluminescent HRP substrate (Millipore, Billerica, MA). The following antibodies were used for western blot: DLC1 monoclonal antibody (BD Transduction Laboratories, San Diego, CA), Anti-V5 (Invitrogen, Carlsbad, CA), α-catenin and IκBα (Santa Cruz Biotechnology, Santa Cruz, CA), NF-κB (p65) and Phospho p65 antibody (Cell-signaling, Danvers, MA), and pIκBα and GFP antibodies (Abcam, Cambridge, MA).

### Immunofluorescence analysis

Cells were fixed in 4.0% paraformaldehyde for 20 min, permeabilized with 0.2% Triton- X for 5 min, and then subjected to staining with following antibodies: Goat polyclonal DLC1, mouse monoclonal anti-E-cadherin, anti-α-catenin, anti-NF-κB (p65) (Santa Cruz Biotechnology, Santa Cruz, CA), and pNF-κB (Cell-signaling Danvers, MA). For detection, anti-mouse Alexa Fluor 488, anti-goat Alexa Fluor568, and anti-mouse Alexa Fluor 650 (Molecular probe, Carlsbad, CA) were used. Stained cells were examined by a Zeiss LSM 510 NLO confocal system (Carl Zeiss Inc, Thornwood, NY, USA) with an inverted microscope (Axiovert 200 M) and a 30 mW argon laser tuned to 488 nm, a 1 mW HeNe laser tuned to 543 nm and a 1 mW HeNe laser tuned to 633 nm. Images were collected using a 63 × Plan-Apochromat 1.4 NA oil immersion objective and a multi-track configuration in Zeiss AIM software (v. 4.0) where the Alexa 488, Alexa 594, and Alexa 633 signals were sequentially collected in separate PMTs with a BP 500–530 nm filter, BP 565–615 nm filter and BP 650–710 filter. Zoom was set at 0.8 and images were 512 × 512 pixels with line averaging of 4.

### Preparation of sub cellular fractions

Subcellular fractions were prepared as described previously (Hodge et al. 2003). Briefly, cells were washed in ice-cold PBS twice, to remove all media, then washed in buffer A (100 mM sucrose, 20 mM HEPES pH 7.4, 1.5 Mm MgCl2, 1 mM EGTA, 1 mM EDTA and 1 mM DTT) and resuspended in 500 μl of buffer B [buffer A plus 5% Percoll, 0.01% digitonin, protease inhibitors cocktails and 1 mM phenylmethylsulfonyl fluoride. After 20 min incubation on ice, the supernatant was centrifuged for 15 min at 15,000 × g to pellet mitochondria. Then supernatant was centrifuged further at 100,000 × g for 1 h. After centrifugation, supernatant and pellet were designated as the cytosolic and membrane fractions, respectively. Membrane pallet was washed twice with Buffer A and then dissolved in appropriate amount of Buffer A supplemented with 1.0% Triton-X.

### RhoA activation assay

RhoA activation assay was done according to manufacturer’s (Cell Biolabs, San Diego, CA) instructions. Briefly, cells were lysed in 1X lysis buffer supplemented with protease inhibitors cocktail (Sigma-Aldrich, St. Louis, MO) and the lysate was centrifuged at 4°C for 10 min at 12,000 × g. An aliquot of lysate was analyzed for total protein content and total RhoA, and the rest of the lysates was used for the Rhotekin assay. Rhotekin beads were added to cell lysates, incubated for 4 hours, and washed thoroughly 3–4 times in washing buffer. Active Rho protein bound to beads was separated in 4-12% acrylamide gels and immunoblotted with anti-RhoA antibody (Abcam, Cambridge, MA).

### Ubiquitination assay

C42-B2 cells were transduced with Ad-LacZ/Ad-DLC1 or transfected with R718E DLC1. Cells were treated for 8 hours with 10 μM of proteasome inhibitor MG132 (Sigma-Aldrich, St. Louis, MO), lysed and the lysates were precleared. Ten micrograms of IκBα antibody were added to 500 μg of cell lysate and incubated in cold room for 4 hours. Then, protein G sepharose was added and incubated for further 2 hours. After centrifugation at 5000 g for 5 min, pellets were washed 3–4 times with lysis buffer, proteins were boiled in loading buffer and subjected to immunoblotting analyses with anti-ubiquitin antibody.

### Colony formation in soft agar

Soft agar assay was performed using Cell Transformation Detection Assay kit (Chemicon, Temecula, CA). Cells were suspended in 0.35% low melting agarose at 2 × 10^4^ cells per well, plated on a layer of 0.8% agarose/T-medium (5%) in six-well culture plates and cultured at 37°C with 5% CO_2_. After 4 weeks, colonies were stained and imaged.

### Apoptosis analysis

Cells were harvested by trypsinization and washed twice with ice-cold PBS. Samples were centrifuged for 3 min at 200 × g, rinsed with PBS, and re-suspended in FITC-labeled annexinV/propidium iodide (PI) according to the manufacturers instructions (Chemicon, Temecula, CA). Apoptotic cells were analyzed by flow cytometry on a FACScan Instrument (Becton Dickinson, San Jose, CA).

### Invasion assay

Cell invasion assays were performed using 8 μm pore-size chambers (Becton Dickinson, Franklin Lakes, NJ). Cells were transduced with Ad-DLC1 or Ad-LacZ or stably transfected with ShRNA of NF-κB or treated with 150 μM of peptide inhibitor of NF-κB (IMGENEX, San Diego, CA) and 1 × 10^6^ cells/well were plated on ECMatrix coated cell culture inserts. After incubation, the non-invading cells were removed from the interior of the inserts using a cotton-tipped swab. Invaded cells were stained with DiffQuick and counted (Dade Behring, Newark, DE, USA).

### Luciferase reporter assay

Ad-LacZ or DLC1 transduced C4-2-B2, and α-catenin transduced PC-3 cells were transfected with NFκB reporter plasmid, or negative and positive control plasmid (SA Biosciences, Frederick, MD) and incubated for 48 hours. Cells were washed with PBS (pH-7.6) and lysed with reporter lysis buffer (Promega, Madison, WI). After centrifugation for 10 mins, supernatant was collected. Luciferase activity was measured using the Dual-Luciferase Reporter Assay System (Promega, Madison, WI).

### Statistical analysis

Statistical analysis of quantitative data was carried out when appropriate, and levels of statistical significance were assessed using two-tailed paired Student’s T- test. All numerical values represent means ± S.E.M from three independent experiments.

### Ethical approval

This research study has been conducted in full compliance with the “Guidelines for the Conduct of Research in the Intramural Research Program at NIH”, issued by the NIH Committee on Scientific Conduct and Ethics, and approved by the NIH Scientific Directors (4th Edition, May 2007).
